# A Case of Puerperal Convulsions in Charge of David Dodge, M. D., of Chicago

**Published:** 1862-05

**Authors:** 


### A CASE OF PUERPERAL CONVULSIONS

IN CHARGE OF DAVID DODGE, M. D., CHICAGO.

Mrs. H., aged about thirty, primapara, at about the seventh month of pregnancy, complained of restlessness and headache. It could not be ascertained that there had been previously during pregnancy, anything out of the usual course. Nine weeks previous had been awakened at night by a severe pain in the head, "as though something had struck her." She is of good figure, and evidently of strong constitution, having never been sick, and by no means of a nervous temperament.

Her husband being about to leave town for a few weeks, requested Dr. D. to call and prescribe for the difficulties above mentioned. Prescribed a mixture of rhubarb, magnesia and aromatic water, to relieve the constipation present. Little or none of it, however, was taken.

Wednesday, at 5 P. M, Jan. 14, the occupants of an adjoining room, heard a sudden fall in the patient's room—on entering, found her on the floor, insensible, and in strong general convulsions. Dr D. was immediately sent for, he arrived in about ten minutes from the access of the convulsions, at which time the convulsive action had ceased, but she remained in a state of insensibility.

Applied rubefacients to the extremities and exhibited an antispasmodic mixture composed of Lac Assafætida, Fl. Ext. Valeriana and Sulph. Ether. Convulsions returned violent in about fifteen minutes. After second convulsions, endeavored to exhibit a cathartic dose, ten grs. of calomel, with ipecac. Very little was swallowed. Convulsions continued recoming about once in fifteen minutes.

Dr. J. A. Allen came in consultation at about 8 o'clock. Exhibited fifteen grs. calomel, with two grs. of ipecac and half a grain of sulph. morphia, enemata of lac assafœtidæ, chloroform inhalations to moderate severity of convulsions.

The intervals of repose gradually lengthened, and the paroxysms became less severe, until between 3 and 4 o'clock A M., when they again became more and more frequent and violent, and on examination it was found that uterine contraction was commencing, the os uteri so patulous as readily to admit the finger. Convulsions continued with fearful severity, apparently very little controlled by the chloroform until about 7 o'clock, when a free evacuation of the bowels, showing powerful influence of the mercurial, took place accompanied with a free discharge of high colored urine, upon which the convulsions became much less frequent and powerful, postponing at one time nearly an hour and a-half.

Uterine contraction tolerably regular once in twenty or thirty minutes, but weak. *Os dilatable.* Ordered Secale Cornut., diffused in water, about ℈ij by enema. The pains became stronger, and the fœtus was expelled about 3 P. M., dead, but not decomposed. It may be remarked in passing, that for two months the movements of the fœtus had been remarkably feeble, so much so as to alarm the mother, who having been a staunch Hydropathist, undertook to strengthen both herself and child by frequent cold baths, after which it appears there was little or no reaction.

At 5 o'clock, P. M., subsequent to delivery, she had another strong convulsion.

Exhibited ten grs. Calomel with a grain of Ipecac, and directed the attendant to stand by the bedside and on the first appearance of restlessness, such as had previsusly preceded a paroxysm, to cause the patient to inhale the Chloroform freely.

The Chloroform prevented the development of the convulsions perfectly, but the inclination continued until a little after midnight, when three full and free evacuations of the bowels taking place, the tendency passed away and she had no further difficulty beyond that ordinarily experienced after a severe labor.

The first time she spoke or showed signs of consciousness, was after the first evacuation of the bowels secured Wednesday morning—full consciousness began to be manifested after the large evacuations Thursday morning, a little after midnight.

She remained more or less disturbed in mind, and dull, for three or four days.

Among the incidental and troublesome symptoms, may be mentioned the immense accumulation of mucus in the throat during Tuesday night and Wednesday, requiring constant attention to prevent its producing mechanical suffocation.

The general plan of treatment adopted in the case, thus briefly sketched, was adopted on the general idea that morbific material was retained in the system, thus by its abnormal presence producing pain, then convulsive phenomena and then insensibility.

The indications were to control the disordered innervation giving rise to the inordinate muscular contraction ; and second, to eliminate the morbific material. Simple anti-spasmodics, then Morphia, and then Chloroform were employed to meet the first indication.

Calomel, believed to be the most potent of known eliminants, was employed to meet the second. Its more speedy general influence was to be favored by the adition of Ipecac.

The cause of Puerperal Convulsions may act primarily, either upon the central ganglia, or at the peripheral distribution. In the former case eliminants are indispensible—in the latter, large reliance may be had upon such agents as the Chloroform and Antispasmodics. Where the cause is clearly peripheral, (Ex-centric,) eliminats are of less importance. Where it is central in action, they are all-important. The sedative influence of blood-letting will occasionally be found useful in ex-centric cases—in centric cases it is clcar, both "theoretically," and (fearfully so) practically, that *it is not a remedy* but something else.

When the cause of the disease operates so profoundly as to give rise to parturifacient contraction, unless anodynes and Chloroform fail to arrest the pains, it is better, when they are tardy, to centralize and intensify the uterine contraction by Secale or some similarly acting ecbolic. Indeed in many cases depending upon excentric causes, the convulsions will cease at once on the supervention of powerful uterine contractions, either spontaneously or by the effect of Ergot. On the other hand, we have seen cases which clearly evinced that Ergot may by irregular action, or when there is continuous mechanical obstruction, positively produce Eclampsia. This is no paradox to a physiologist with brains.

When time will admit, other eliminants may be employed, and especially dierutics, at the head of all which latter still stands the Acetate of Potash. In the present case owing to the great difficulty in swallowing but little could be used, and the main reliance was placed in *old* Calomel and *young* Chloroform.

